# Draft Genome Sequence of *Agarivorans* sp. Strain Toyoura001, Isolated from an Abalone Gut

**DOI:** 10.1128/MRA.00169-19

**Published:** 2019-05-09

**Authors:** Hideomi Itoh, Keisuke Kawano, Minoru Kihara

**Affiliations:** a Bioproduction Research Institute, National Institute of Advanced Industrial Science and Technology (AIST), Sapporo, Japan; b Department of Marine Biology and Sciences, School of Biological Sciences, Tokai University, Sapporo, Japan; University of Southern California

## Abstract

Agarivorans sp. strain Toyoura001 is a bacterium isolated from the gut of a wild abalone, Haliotis discus hannai. Here, we report the draft genome sequence of strain Toyoura001, which consists of 60 contigs comprising 4.67 Mb and 4,257 protein-coding genes.

## ANNOUNCEMENT

*Agarivorans* spp. are marine bacteria that have been isolated from various marine animal and environmental sources (1–4). One of the common phenotypic features among *Agarivorans* spp. is that they can degrade seaweed polysaccharides (5–7). Recently, we isolated *Agarivorans* sp. strain Toyoura001 from the gut of a wild abalone, *Haliotis discus hannai*. Abalones are well-known marine herbivores that feed on seaweed; however, their digestive ability is not strong enough to completely degrade seaweed polysaccharides, suggesting a supportive role of their gut bacteria for polysaccharide degradation (8). To facilitate the future characterization of the association of Toyoura001 with abalone, we report here the draft genome sequence of this bacterium.

The gut contents of the abalone were spread onto a medium containing marine broth 2216 (Difco Laboratories) and 1.5% agar. After incubation at 15°C for 10 days, the strain Toyoura001, which formed white colonies with a dent, was isolated. A partial sequence (1,433 bp) of its 16S rRNA gene was identified as described previously (9), and it exhibited the closest similarity (98.19%) to that of Agarivorans albus MKT106^T^ according to a BLASTn search against the NCBI database, indicating that Toyoura001 belongs to the genus *Agarivorans*.

The genomic DNA of Toyoura001 was extracted by following general protocols (10). The integrity of the extracted DNA was determined using a NanoDrop ND-1000 spectrophotometer (NanoDrop Technologies) and QuantiFluor double-stranded DNA (dsDNA) system (Promega). After the fragmentation of 500 bp of the purified DNA using an M220 instrument (Covaris), a DNA library was prepared using the HyperPrep kit (Kapa Biosystems), according to the manufacturer’s instructions. The integrity of the prepared library was confirmed on a Bioanalyzer (Agilent Technologies). Paired-end sequencing (2 × 151 bp) of the prepared library was performed on a NextSeq sequencer (Illumina); 1,606,264 sequences were obtained. Low-quality (with a Q score of <20) and short-length (<128-base) sequences were trimmed and discarded using Sickle ver. 1.33 (11); the remaining 1,287,916 sequences were then assembled *de novo* using SPAdes ver. 3.10.1 (12), with the “careful” mode enabled and k-mer sizes of 21, 33, 55, 77, 99, and 127. The resulting draft genome of Toyoura001 was annotated using the DDBJ Fast Annotation and Submission Tool (DFAST) ver. 1.1.0 with default settings (13). The genes encoding glycoside hydrolases (GHs), polysaccharide lyases (PLs), and carbohydrate-binding modules (CBMs), were detected from an HMMER search (cutoff; E value, <1.0 × 10^−15^; coverage, >0.35×) against the Carbohydrate-Active enZYmes (CAZyme) database on the dbCAN2 server (14).

The assembled genome of *Agarivorans* sp. Toyoura001 consisted of 60 contigs with a length of >1,000 bp (longest contig, 706,345 bp; *N*_50_, 186,035 bp; 83× genome coverage); it comprised 4,666,209 bp and had a G+C content of 44.2%. Annotation with DFAST predicted genes for 4,257 protein-coding sequences, 10 rRNAs and 59 tRNAs. The genome encoded diverse enzymes involved in the hydrolysis of polysaccharides that are abundant in seaweeds, such as agar (GH16, GH50, GH86, and CBM16), alginate (PL6, PL7, PL14, PL15, and PL17), and carrageenan (GH16 and CBM16). This suggests that Toyoura001 could potentially degrade seaweed-derived polysaccharides.

### Data availability.

The partial sequences of the 16S rRNA gene and the genomic sequences of Toyoura001 have been deposited in the DDBJ/ENA/GenBank under the accession numbers LC414627 (the version described here is the first version, LC414627.1) and BJEQ00000000, respectively. Raw genomic sequencing data have also been deposited under the accession number DRX137605.
